# Divergent Response of Host‐Specific Driven Gut Microbial Stability in Freshwater Gastropods to Cyanobacterial Blooms

**DOI:** 10.1002/ece3.72541

**Published:** 2025-11-18

**Authors:** Kexin Meng, Wen Yang, Shuangye Pan, Kaihong Lu, Jinyong Zhu

**Affiliations:** ^1^ School of Marine Sciences, Ningbo University Ningbo China; ^2^ Zhejiang Ningbo Ecological and Environmental Monitoring Center Ningbo China

**Keywords:** community stability, cyanobacteria bloom, gastropod, gut microbiota, keystone taxa

## Abstract

Gut microbial community stability is critical for host environmental adaptation. Cyanobacteria have been shown to disrupt the gut microbial composition, which can lead to gut microbiota dysbiosis. However, little is known about the effects of cyanobacteria on the stability of gut microbial communities in different hosts. In this study, two freshwater gastropods (*Bellamya aeruginosa* and *Pomacea canaliculata* ) were used as controls to study the differences in the effects of cyanobacteria on their gut microbial communities in ponds with the absence of cyanobacterial blooms. Cyanobacterial blooms cause distinct differences in the diversity, community structure, and assembly mechanisms of gut microbiota between the two freshwater gastropod species. The gut microbiota of *B. aeruginosa* exhibits significantly lower robustness (*p* < 0.01) and greater vulnerability under cyanobacterial exposure, indicating reduced community stability, whereas *canaliculata* exhibits greater adaptive capacity. Microbial co‐occurrence networks suggest that cyanobacteria influence potential interactions within the gut microbial community of *B. aeruginosa*, and that the number of potential keystone taxa that maintain community stability is low in water bodies with occurrences of cyanobacterial bloom. This study reveals the differential response of the gut microbial community stability to cyanobacterial blooms in different snail hosts, providing new perspectives for understanding the adaptation mechanisms of gastropods to environmental stresses.

## Graphical abstract

In this study, two freshwater gastropods (Bellamya aeruginosa and *Pomacea canaliculata* ) were used as controls to study the differences in the effects of cyanobacteria on their gut microbial communities in ponds with the absence of cyanobacterial blooms. Cyanobacterial blooms cause distinct differences in the diversity, community structure, and assembly mechanisms of gut microbiota between the two freshwater gastropod species. This study reveals the differential response of the gut microbial community stability to cyanobacterial blooms in different snail hosts.

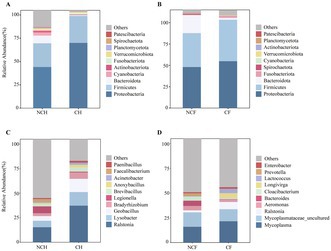

## 1 Introduction

Cyanobacterial blooms, catastrophic ecological phenomena in freshwater ecosystems caused by the massive proliferation and accumulation of cyanobacteria, have aroused widespread concern worldwide (Paerl and Otten 2013). These cyanobacteria produce a variety of cyanotoxins, including microcystins (MCs), which accumulate through the food chain and pose a significant threat to aquatic life and human health (Pavagadhi and Balasubramanian 2013). Freshwater gastropods are more susceptible to MCs because of the temporal coincidence of their breeding season with the high incidence of blooms (Lance et al. 2010). MCs enter the body of gastropods through the intestine or gills and accumulate in different tissues, causing unfavorable physiological responses to individual organisms that ultimately lead to changes in the structure of the gastropod community (Gérard et al. 2008). As a key species of benthic ecosystems, snails play an irreplaceable role in the decomposition of organic matter, material cycling, and maintenance of biodiversity (Ma et al. 2010). Changes in the gastropod community structure caused by cyanobacterial blooms and their toxins will directly affect energy flow efficiency, cause a reduction in the value of water ecosystem services, and lead to a decline in biodiversity.

In recent years, with the development of microbiomics profiling technology, the snail gut microbial community is strongly influenced not only by host‐related factors (e.g., species (Hu et al. 2024) and developmental stage (Chen, Li, et al. 2021)) but also by the environment, and factors such as food, habitat alteration (Walters et al. 2022; Kang et al. 2022), and stimulation by environmental stress (Wu et al. 2022) not only lead to changes in the composition of the microbial community directly but also disrupt microbiota homeostasis, ultimately leading to functional dysbiosis (Peng et al. 2019). There are complex interactions between microorganisms that can form networks of interconnected ecosystems. After being disturbed by the environment, they can restore the original community state to maintain the stability of the structure and function of the microbial community (Fassarella et al. 2021). Hosts in poor health are often associated with unstable flora (Xiong et al. 2015), so microbiomes that can maintain homeostasis or are resilient in the face of environmental change tend to be highly important for host survival (Sha et al. 2022). Although microbiome stability is important for host homeostasis, it has also been shown that microbiome flexibility can provide the host with enhanced adaptive capacity to cope with environmental changes (Ren et al. 2023). Thus, microbiome stability may be an important marker of the host's adaptive response to environmental change. However, most of the studies have focused on species richness and abundance, while the stability of gut microbial communities and their maintenance mechanisms remain critically underexplored. Whereas the host can also influence these response patterns, studies of gastropod symbioses in community stability under environmental perturbations are particularly lacking.

*Bellamya aeruginosa* is a native freshwater snail widely distributed in East Asia and plays a key role in structuring aquatic ecosystems (Zhijun et al. 2009). Previous studies have shown that this species exhibits relatively high sensitivity to different types of contaminants (Yao et al. 2020; Han et al. 2024), rendering it an ideal bioindicator for assessing the ecotoxicity of contaminated sediments. *Pomacea canaliculata* is a common exotic freshwater gastropod. It has been recognized as one of the most successful invasive species because of its high tolerance to intense environmental stresses, high fecundity, rapid growth rate, and complex diet, causing significant damage to biodiversity and ecosystem functioning (Martín et al. 2019). Both species of snails are commonly found in water bodies with algal blooms; however, there are significant differences in the accumulation of MCs in their tissues (Trung et al. 2018; Wang et al. 2023), Notably, only limited studies have reported the effects of toxic cyanobacterial blooms on the gut microbial composition and functioning of *B. aeruginosa* (Yang et al. 2023), and the effects of a toxic cyanobacterial bloom on the gut microbiota of *P. canaliculata* have not been reported.

In this study, two freshwater gastropods (*B. aeruginosa* and *P. canaliculata* ) coexisting in the same habitat were selected to assess differences in the structure and stability of their gut microbial communities in water bodies with occurrences of cyanobacterial bloom (WCB) and in water bodies with the absence of cyanobacterial bloom (WACB). We wanted to address two main questions: (1) whether there are differences in the composition, diversity, community assembly, and stability of gut microbial communities between the two snails in response to cyanobacteria; (2) whether specific keystone taxa play an important role in the stability of gut microbial communities in both snails.

## 2 Materials and Methods

### 2.1 Collection of Samples

From May to November 2023, samples of *B. aeruginosa*, *P. canaliculata* , and water were collected from the littoral zone of a pond with occurrences of cyanobacterial bloom in Ningbo, Zhejiang Province, with a pond area of approximately 0.35 ha and a depth of approximately 1.5 m (29°54′42.318″N; 121°38′49.497″ E). Although the pond has undergone several ecological restorations (dredging and macrophyte restoration), cyanobacterial blooms dominated by *Microcystis* continue to occur regularly. Two snail species of samples and water samples were collected from another pond with the absence of cyanobacterial bloom, with a pond area of approximately 12 m^2^ and a depth of approximately 0.9 m (29°54′50.982″N; 121°38′31.840″ E). The collected snail samples were placed in plastic containers with sterile water and returned to the laboratory. After scrubbing the surface of the snails with 70% ethanol, the shells were crushed, dissected, isolated, and removed from the intestine and hepatopancreas for gut microbiota analysis and MCs content analysis, respectively. To obtain representative samples and to reduce individual differences, tissues from three individuals of the same snail species were pooled and processed as replicate samples for subsequent analysis. Each sample has three replicates.

### 2.2 Phytoplankton Analysis

Phytoplankton samples (approximately 250 mL) were obtained monthly from ponds and immediately fixed in 1% Lugol iodine solution. By the quantitative Utermöhl method, phytoplankton were identified using a 50 mL tubular sedimentary phytoplankton counting frame under an inverted microscope (CK2, Olympus, Tokyo, Japan) at 1000× magnification (Utermöhl 1958). Phytoplankton biomass was volumetrically calculated with OptiCount software (SequentiX, Klein Raden, Germany). The specific density of the phytoplankton cells was assumed to be 1 g/cm^3^ (wet weight).

### 2.3 Measurement of Physicochemical Factors and MCs

Basic physicochemical indicators, such as pH, water temperature (WT), and dissolved oxygen (DO), were measured in situ via a multifunctional water quality tester (YSI6000; YSI Inc., Yellow Springs, USA). Secchi depth (SD) was estimated via a Secchi disk. Chlorophyll a (Chl‐a) was measured in the water samples via the hot ethanol method (Chen et al. 2006). Water samples were analyzed for chemical components, including nitrite nitrogen (NO_2_‐N), nitrate nitrogen (NO_3_‐N), ammonium nitrogen (NH_4_‐N), total nitrogen (TN), orthophosphate (PO_4_‐P), and total phosphorus (TP), quantitatively via a SmartChem 2000 (Westco Scientific Instruments, Brookfield, USA) discrete spectrophotometer.

The hepatopancreas was weighed before and after lyophilization, and MCs were extracted according to the method of Yang et al. (2022). An enzyme‐linked immunoassay (ELISA) kit was used to determine the concentration, and the detection range of microcystins was 0.1–2 μg/L (Beacon Analytical Systems, Portland, ME, USA), since the kit cannot be used to identify variants of microcystins. Therefore, the concentration of MCs was expressed as the equivalent of MC‐LR. The recovery and matrix effects of the extracted MCs were determined by adding an MC‐LR standard (3 μg/g dry weight, purity > 98%; MedChemExpress, Monmouth Junction, NJ, USA) (Zhang et al. 2016). The extraction efficiency of MCs was 91.6% when the same amount of 100% methanol was added. The matrix effect on the hepatopancreas was negligible. The high recovery of MCs extraction and the low matrix effect of the assay allowed this study to obtain MCs directly without additional calculations.

### 2.4 DNA Extraction, Amplification, and Sequencing

Snail intestinal genomic DNA was extracted from individual snails using a DNA extraction kit (Minka Gene Bacterial DNA Kit) according to standard DNA extraction procedures, which were performed according to the manufacturer's instructions. The V3–V4 variable region of the 16S rRNA gene was amplified using primers 338F (5′‐ACTCCTACGGGGAGGCAGCA‐3′) and 806R (5′‐GGACTACHVGGGGTWTCTAAT‐3′). The PCR amplification program was set as follows: 95°C for 3 min, 95°C for 30 s, 50°C for 30 s, and 72°C for 45 s, with the final extension required at 72°C for 10 min. A PCR fragment purification kit from Takara Biotech (Japan) and a Quant‐iT Pico Green dsDNA quantification kit from Invitrogen (USA) were used for purification and quantification analysis. Purification and quantification were performed via QuantiFluor‐ST (Promega, Madison, WI, USA), after which the products were mixed in equimolar concentrations and finally sequenced on the Illumina MiSeq platform (Caporaso et al. 2012).

Using USEARCH (version 11) to merge paired‐end sequence reads. The paired reads were merged with the fastq_mergepairs function and filtered using a “maxee” value of 1.0. Unique sequence reads were obtained using the fastx_uniques function, followed by error correction via the UNOISE3 algorithm (unoise_alpha = 4, minsize = 6) for denoising. Remaining sequences were then filtered to remove chimeras and clustered into Zero‐radius Operational Taxonomic Units (ZOTUs) at 97% or higher similarity. All quality‐filtered reads were subsequently mapped to these ZOTUs (Edgar 2016). Representative sequences of each ZOTU were assigned to taxonomic groups using the RDP classifier against the SILVA database (16s_v138), with clustering performed at 99% similarity.

### 2.5 Statistical Analysis

Principal coordinate analysis (PCoA) was performed using the R package “vegan” via the Bray–Curtis distance matrix to explore differences in the community structure of the gut microbial communities (Dixon 2003). A ranked multivariate analysis of variance (PERMANOVA) based on the Bray–Curtis distance was performed to assess the effect of cyanobacterial blooms on the microbial composition of the gut using the ANOSIM function of the R package “vegan” (Dixon 2003). To assess gut microbial diversity, the alpha diversity index (Shannon, Simpson, Pielou, and Chao) was calculated. We calculated the mean nearest taxon distance metric using the “picante” R software package (Kembel et al. 2010), and implemented a previously developed zero‐modeling approach to calculate the Beta‐Nearest Taxon Index (βNTI) to infer community assembly processes (Shade et al. 2012). βNTI > 2 was considered to be the result of deterministic process variable selection with significantly higher than expected phylogenetic turnover rates. βNTI ≤ 2 is considered to be the result of homogeneous selection for deterministic processes, with significantly lower than expected phylogenetic turnover rates. Values of βNTI less than 2 indicate that differences in phylogenetic composition are the result of stochastic processes (Shade et al. 2012).

### 2.6 Network Construction and Stability Analysis

A protozoan community co‐occurrence network was constructed on the basis of the Spearman correlation matrix to assess species interactions. OTUs with relative abundances higher than 0.1% and present in more than 1/3 of all samples were screened for subsequent analysis. Robust correlation thresholds, namely, an absolute correlation coefficient *ρ* > 0.6 and *p* < 0.01, were used to construct the co‐occurrence network (de Araujo et al. 2018). The networks were analyzed and visualized using Gephi software v.0.9.2 (https://www.gephi.org/) using undirected networks and Fruchterman–Reingold layouts. The relevant topological parameters, which represent the topological structure and node characteristics of the network, were obtained using the Gephi platform, including the number of nodes, number of edges, degree, path length, diameter of the network, graph density, clustering coefficients, modularity, and average betweenness centrality and closeness centrality.

To assess the stability of the microbial networks, we calculated the robustness, vulnerability and cohesion of each network. To test the effect of species removal on the remaining species, the abundance‐weighted average interaction strength of the nodes was calculated (Deng et al. 2012). The robustness of this study was considered when 50% of the nodes were randomly deleted, and the results were based on 100 repetitions of the simulation. To assess the speed of interference propagation within the network, the global efficiency is considered the average of all node pair efficiencies, which is calculated as the number of edges in the shortest path between pairs of nodes (Stentiford et al. 2020). The vulnerability reflects the relative contribution of each node to the global efficiency, represented by the maximum vulnerability of the nodes in the network.

Cohesion is a recently developed metric that quantifies community complexity (Hernandez et al. 2021). The negative: positive cohesion (N: P cohesion) corresponding to competitive and cooperative interactions for each community was calculated to quantify microbial connectivity (Herren and McMahon 2017). In general, higher negative cohesion or lower positive cohesion indicates a more stable ecological network. The N: P cohesion value for each sample was calculated via the following formula:

$$\text{conhesion}=\sum_{i=1}^{m}{\text{abundance}}_{i}\times {\text{connectedness}}_{i}$$

where *m* is the total number of taxa in the community. The data were visualized via GraphPad Prism 9.5.

The values of within‐module connectivity (Zi) and among‐module connectivity (Pi) are calculated to reflect the function of the nodes in the network. On the basis of the Zi and Pi values, all nodes are categorized into four groups: module hubs (Zi ≥ 2.5, Pi < 0.62), network hubs (Zi ≥ 2.5, Pi ≥ 0.62), connectors (Zi < 2.5, Pi ≥ 0.62), and peripherals (Zi < 2.5, Pi < 0.62). Modular hubs, network hubs and connectors are often recognized as potential keystone taxa for maintaining network stability (Stentiford et al. 2020). Finally, the contribution of individual keystone taxa (OTUs) to community stability was predicted via random forest analysis. To estimate the importance of these diversity indices, we used the percentage of MSE (mean square error) of the variable: a higher MSE% value means that the variable is more important (Breiman 2001). The significance of each predictor variable for the response variable was assessed via the “rfPermute” package.

### 2.7 Piecewise Structural Equation Modeling (SEM)

To further explore the influence of the environment on community stability, we used piecewise structural equation modeling (SEM) to infer the direct and indirect ways in which the environment affects community stability. Before modeling, the best combination of environmental variables is selected via a forward selection procedure via “ordiR2step ().” All piecewise SEM was performed via the R package “piecewiseSEM” (Lefcheck 2016).

## 3 Results

### 3.1 Aquatic Physicochemical Factors and Phytoplankton Community Dynamics

The WT in the pond with the absence of cyanobacterial bloom increased gradually from May until it peaked in August (29.8°C) and then declined (Table S1). pH and DO varied in the ranges of 6.85–7.95 and 4.13–9.32 mg/L, respectively. SD could not be measured because of the shallow depth of the present body of water. NH_4_‐N, NO_3_‐N, and TN showed similar trends, with ranges of 0.09–0.24 mg/L, 0.302–0.924 mg/L, and 0.682–1.152 mg/L, respectively. PO_4_‐P and TP showed similar trends, with the lowest values occurring in November, at 0.054 mg/L and 0.069 mg/L, respectively. The trend of the WT changes in the pond with occurrences of cyanobacterial blooms was the same as that in the pond with the absence of cyanobacterial bloom (Table S1). pH, DO, and SD varied in the ranges of 6.23–8.51, 1.49–9.14 mg/L, and 0.17–0.71 m, respectively. NH_4_‐N, NO_2_‐N, NO_3_‐N, and TN followed similar trends of decreasing and then increasing, with the lowest values occurring in July, at 0.04, 0.02, 0.28, and 0.61 mg/L, respectively. PO_4_‐P and TP showed similar trends, with ranges of 0.05–0.15 mg/L and 0.06–0.83 mg/L, respectively.

In the pond with the absence of cyanobacterial bloom, 38 species of phytoplankton belonging to 6 phyla were identified, including 23 species of Chlorophyta, 6 species of Euglenophyta, 3 species of Bacillariophyta, 3 species of Cyanophyta, 2 species of Pyrrophyta, and 1 species of Cryptophyta. There were significant differences in the biomass of different phytoplankton phyla in different months, with Pyrrophyta having higher biomass in July and August, while Chlorophyta had the highest biomass in October (Table S2). In the pond with occurrences of cyanobacterial bloom, 52 species of phytoplankton belonging to 7 phyla were identified, including 25 species of Chlorophyta, 9 species of Cyanophyta, 8 species of Bacillariophyta, 5 species of Euglenophyta, 2 species of Pyrrophyta, 2 species of Cryptophyta, and 1 species of Chrysophyta. Severe cyanobacterial blooms were present in the ponds beginning in May and continuing through November, with the biomass share of cyanobacteria exceeding 50% in May, June, August, and September (Table S2). Notably, we also identified a variety of cyanobacterial bloom species such as *Microcystis aeruginosa* , *Oscillatoria subbrevis,* and *Anabaena circinalis* , with *M. aeruginosa* being overwhelmingly dominant.

### 3.2 Microbial Diversity and Composition of the Snail Gut

**FIGURE 1:**
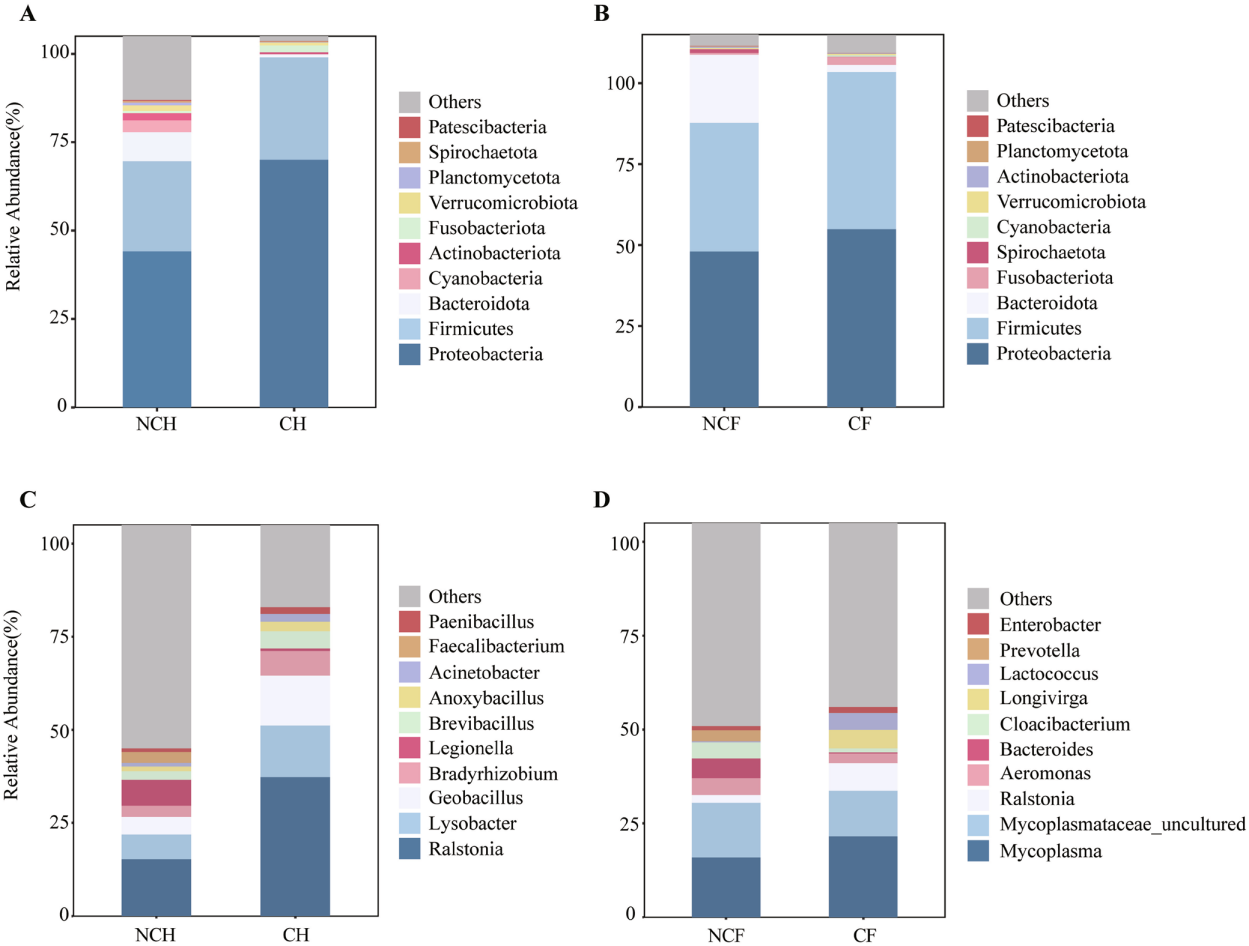
Relative abundance of *B. aeruginosa* gut microbial communities at the phylum (A) and genus levels (B) and *P. canaliculata* gut microbial communities at the phylum (C) and genus levels (D) in WCB and WACB. NCH: *B. aeruginosa* in WACB (*n* = 21); CH: *B. aeruginosa* in WCB (*n* = 21); NCF: *P. canaliculata* in WACB (*n* = 21); CF: *P. canaliculata* in WCB (*n* = 21).

The gut microbial amplicon sequences were clustered into 18,472 OTUs. We compared the composition of the gut microbial communities of *B. aeruginosa* and *P. canaliculata* in WCB and WACB at the phylum level (Figure 1A,B). There were significant differences in the composition of the gut microbial communities of *B. aeruginosa* in WACB (NCH) and WCB (CH) (Figure 1A). Among the NCH gut microbiota, Proteobacteria and Firmicutes were the major microbial communities, accounting for 42% and 24%, respectively. In addition, higher abundances of Bacteroidota, Cyanobacteria, and Actinobacteria were also present. In contrast, the microbial community structure of the CH gut microbiota was significantly different, with relatively high relative abundances of Proteobacteria (67%) and Firmicutes (28%). For *P. canaliculata* , Proteobacteria and Firmicutes dominated the gut microbial community of *P. canaliculata* in WACB (NCF) and WCB (CF) (Figure 1B). A similar pattern was also observed at the genus level, where the composition of the gut microbial communities of the two snail species differed markedly (Figure 1C,D). The dominant genera in the gut microbiota of *B. aeruginosa* were *Ralstonia*, *Lysobacter*, and *Geobacillus*, and the abundances of these genera were higher in the CH gut microbiota than in the NCH gut microbiota. The dominant genera in the intestine of *P. canaliculata* were *Mycoplasma* and *Mycoplasmataceae_uncultured*, and these genera were not significantly different in the NCF and CF gut microbiota.

**FIGURE 2:**
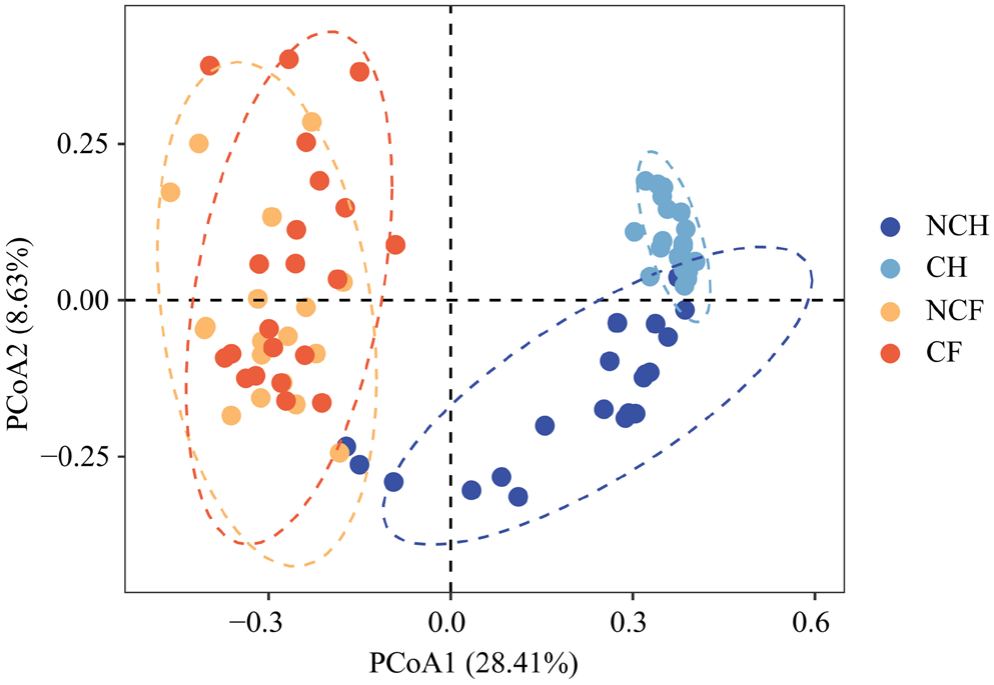
β‐Diversity of gut microbial communities of *B. aeruginosa* and *P. canaliculata* in WCB and WACB was measured by principal coordinate analysis (PCoA) based on the Bray–Curtis distance principle. NCH: *B. aeruginosa* in WACB (*n* = 21); CH: *B. aeruginosa* in WCB (*n* = 21); NCF: *P. canaliculata* in WACB (*n* = 21); CF: *P. canaliculata* in WCB (*n* = 21).

**FIGURE 3:**
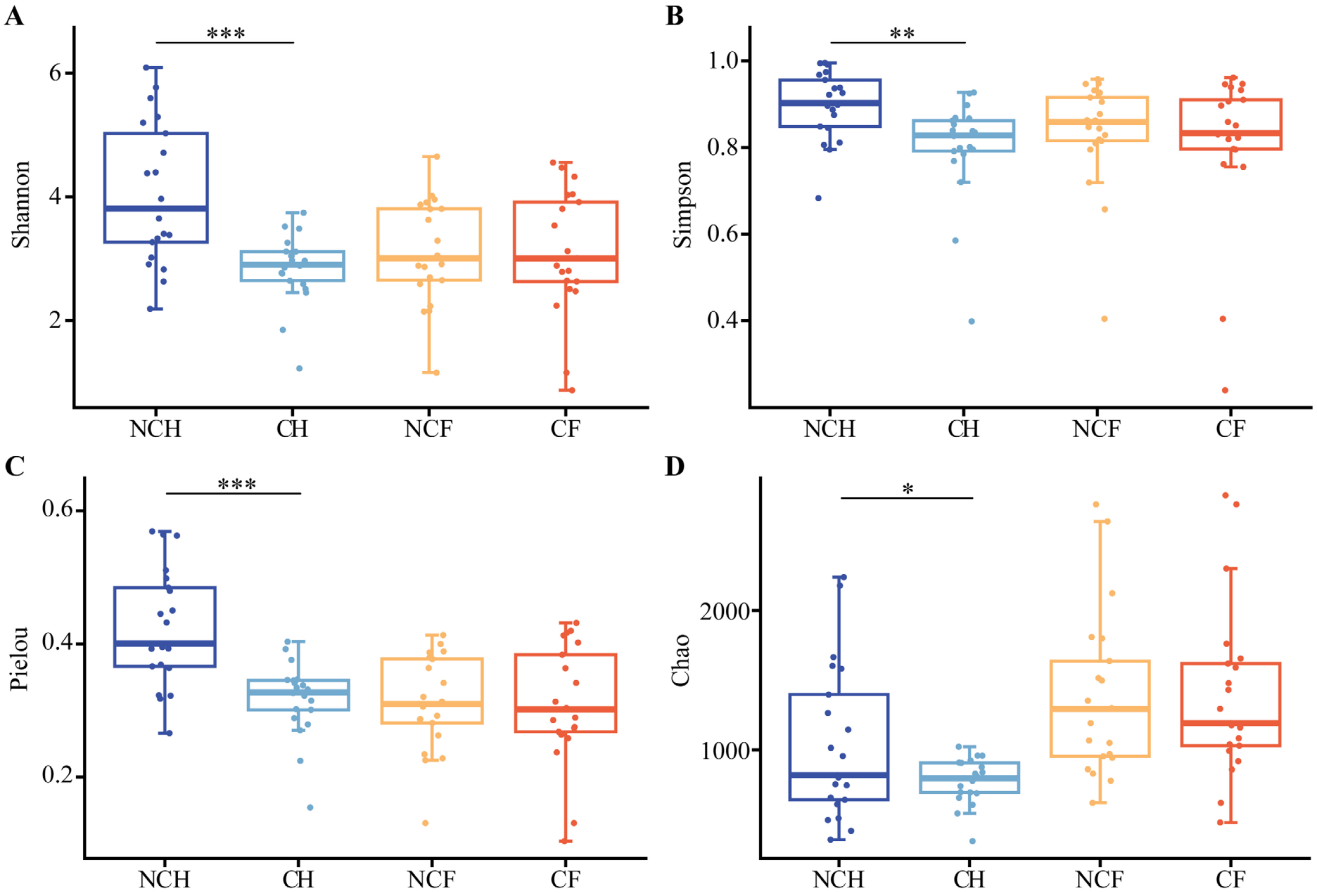
Alpha diversity indices of the gut microbial communities of *B. aeruginosa* and *P. canaliculata* in WCB and WACB. (A) Shannon index, (B) Simpson index, (C) Pielou index, and (D) Chao index. A *t*‐test was used to test for significant differences in gut microbial biology. *p < 0.05; **p < 0.01; ***p < 0.001. NCH: *B. aeruginosa* in WACB (*n* = 21); CH: *B. aeruginosa* in WCB (*n* = 21); NCF: *P. canaliculata* in WACB (*n* = 21); CF: *P. canaliculata* in WCB (*n* = 21).

PCoA based on Bray–Curtis distances revealed significant differences between the NCH and CH gut microbiota, whereas there were no significant differences in the structure of the NCF and CF gut microbiota (*R* ^2^ = 0.33, *p* = 0.001; Figure 2, Table S3). The *α* diversity of the NCH gut microbiota was significantly higher than that of the CH gut microbiota (*p* < 0.01, Figure 3). In contrast, the alpha diversity of the gut microbiota of *P. canaliculata* did not differ significantly between the two water bodies (*p* > 0.05, Figure 3). Overall, there were differences in the diversity of the gut microbiota of *B. aeruginosa* under different conditions. In contrast, the gut microbiota of *P. canaliculata* did not differ significantly.

### 3.3 Assembly of the Gut Microbiota Community in Snails

**FIGURE 4:**
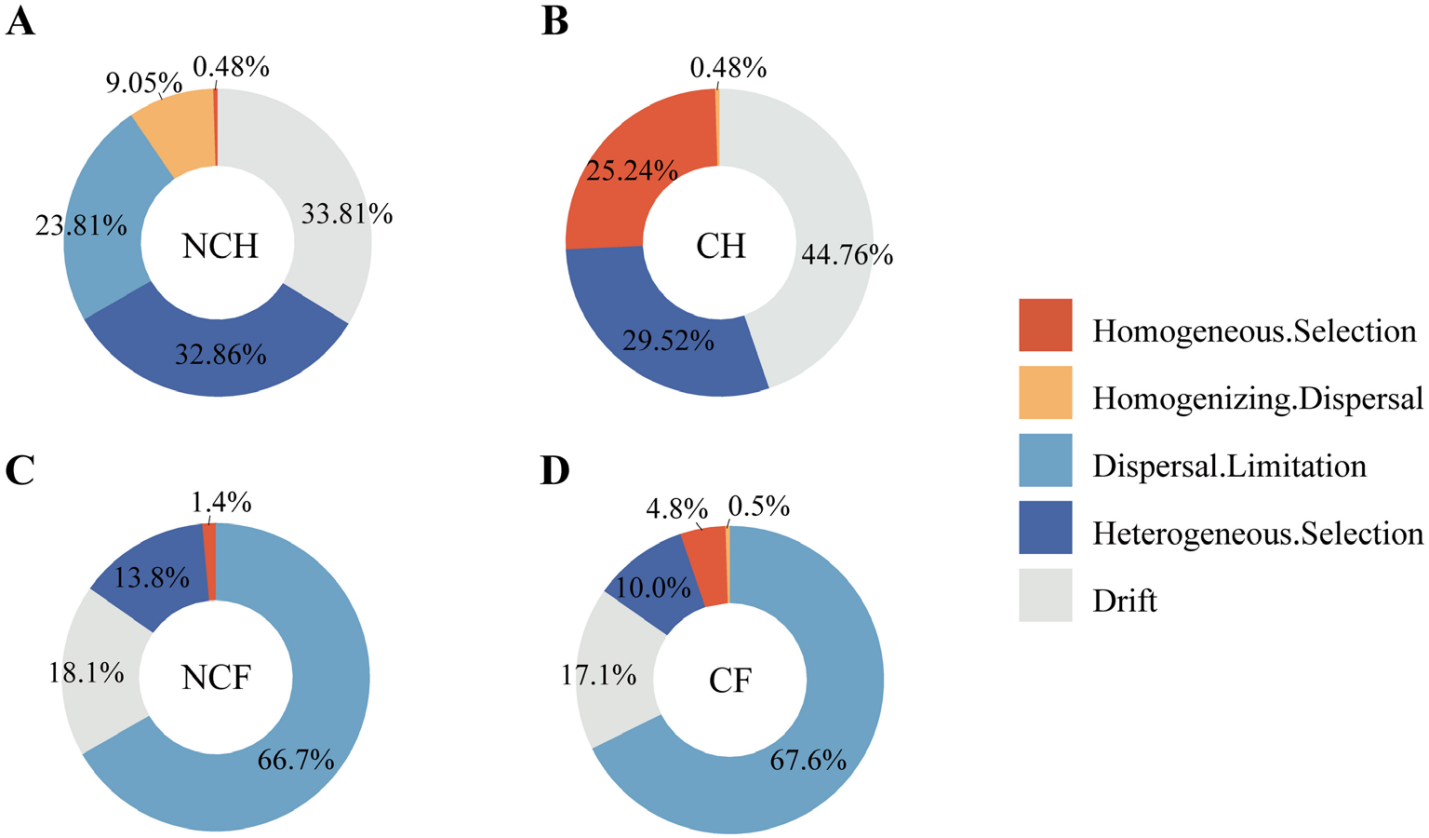
Ecological processes of gut microbial communities of *B. aeruginosa* (A, B) and *P. canaliculata* (C, D) in WCB and WACB analyzed based on null modeling. NCH: *B. aeruginosa* in WACB (*n* = 21); CH: *B. aeruginosa* in WCB (*n* = 21); NCF: *P. canaliculata* in WACB (*n* = 21); CF: *P. canaliculata* in WCB (*n* = 21).

The null model was applied to analyze the assembly processes of the gut microbiota in *B. aeruginosa* and *P. canaliculata* (Figure 4). The results revealed that in the NCH gut microbiota, drift constituted the largest proportion (33.81%), followed by heterogeneous selection (32.86%) and dispersal limitation (23.81%). In the CH gut microbiota, drift dominated (44.76%), with heterogeneous selection accounting for 29.52%. Both the NCH and CH gut microbiotas were subjected to a combination of drift and heterogeneous selection, indicating that the assembly of their microbial communities is governed primarily by stochastic processes and environmental selection. However, the deterministic process of homogeneous selection was significantly higher in the CH gut microbiota than in the NCH gut microbiota. In contrast, the NCF and CF gut microbiota were predominantly driven by stochastic processes, with dispersal limitation (67.6% and 66.7%) and drift (18.1% and 17.1%) as the dominant factors.

### 3.4 Network Complexity of the Gut Microbiota in Snails

**FIGURE 5:**
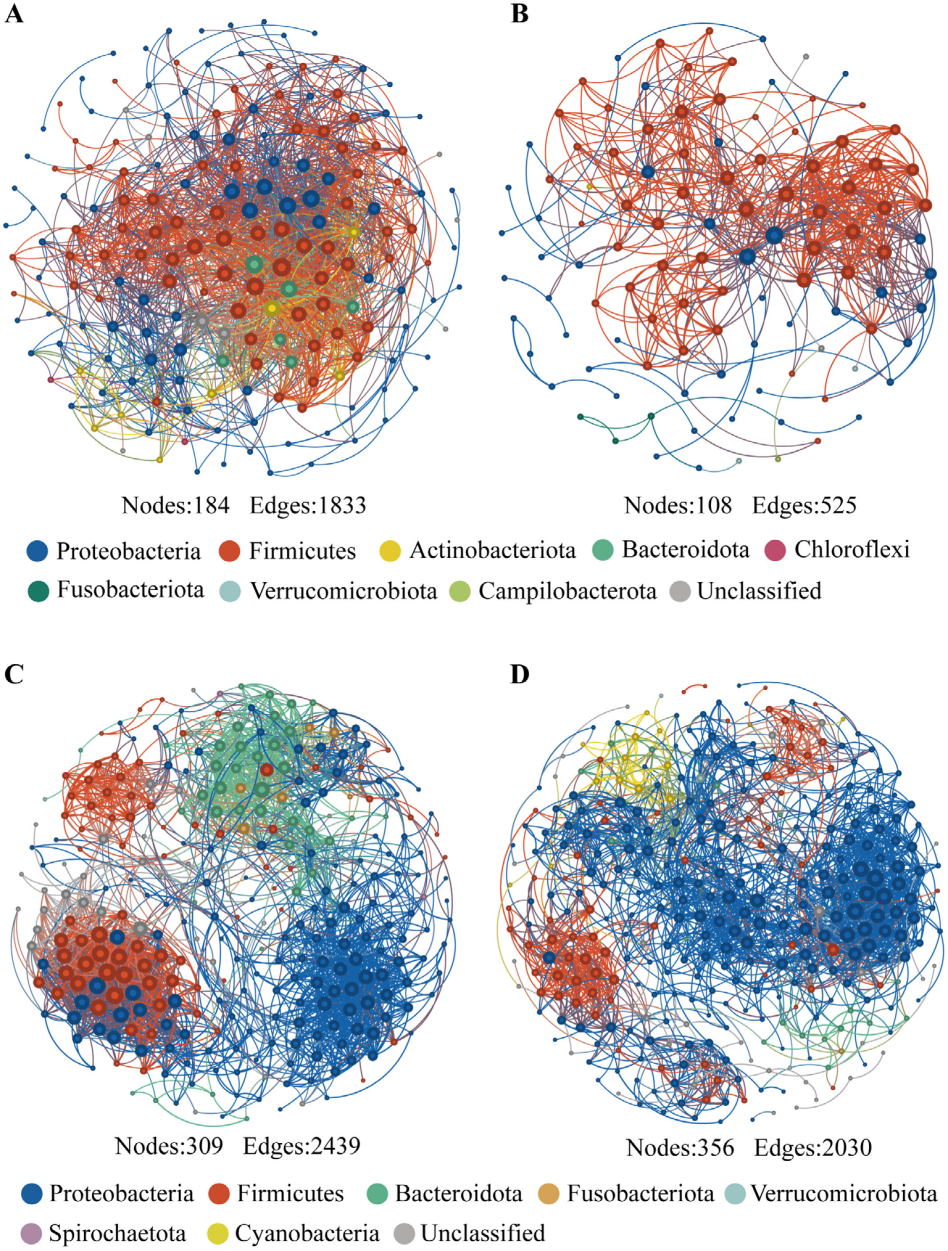
Networks of the gut microbial communities of *B. aeruginosa* (A) and *P. canaliculata* (C) in WACB and *B. aeruginosa* (B) and *P. canaliculata* (D) in WCB. Nodes denote individual OTUs, and the size of the node denotes the degree. The gut microbiota of two snail species in both water bodies comprised 21 samples (*n* = 21).

The co‐occurrence network revealed significant differences in the topological characteristics of the gut network of *B. aeruginosa* in different environments (Figure 5, Table S4). In contrast to the network of the CH gut microbiota, the network of the NCH gut microbiota presented higher connectivity (con), a greater degree of averaging, and a higher number of edges and nodes, suggesting a high level of complexity in its gut microbial network. For the gut network of *P. canaliculata* , the network of NCF gut microbiota presented 309 nodes and 2439 edges, and the network of CF gut microbiota presented 356 nodes and 2030 edges (Figure 5C,D), suggesting that the network complexity of the gut microbial community of *P. canaliculata* was similar under both water conditions.

### 3.5 Network Stability of the Gut Microbiota in Snails

**FIGURE 6:**
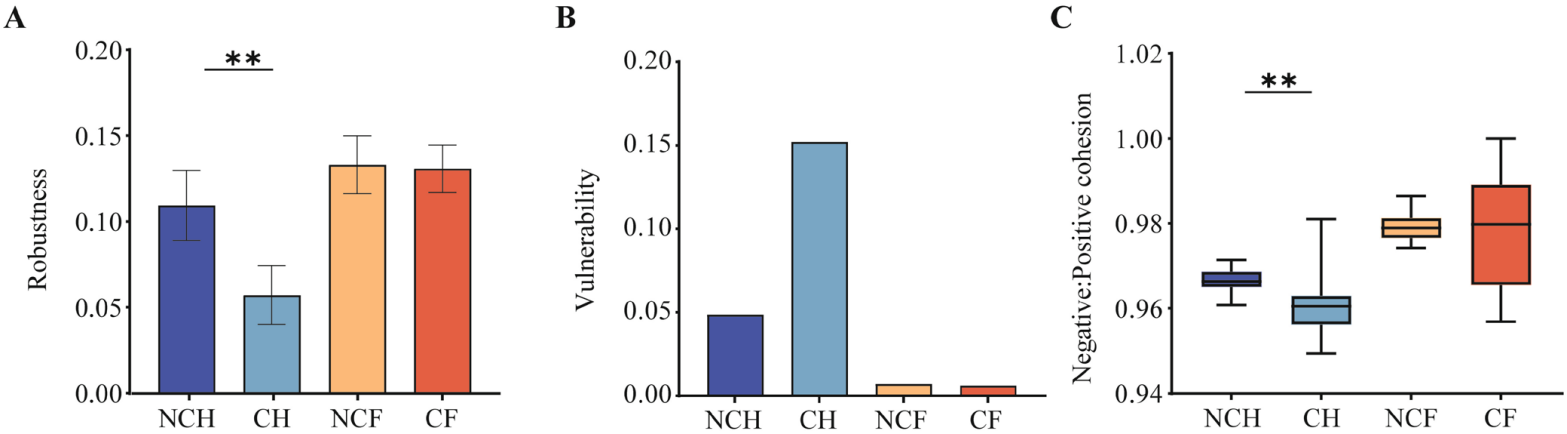
Stability analysis of the gut microbial network. (A) Robustness is measured as the proportion of species remaining in the community after 50% removal of random nodes. (B) The maximum node vulnerability in each community measures vulnerability. (C) Negative: Positive cohesion is calculated from species abundance. ** indicates significant differences (paired Student's *t*‐test, *p* < 0.01). NCH: *B. aeruginosa* in WACB (*n* = 21); CH: *B. aeruginosa* in WCB (*n* = 21); NCF: *P. canaliculata* in WACB (*n* = 21); CF: *P. canaliculata* in WCB (*n* = 21).

The robustness of the NCH gut microbial network was significantly higher than that of the CH gut microbial network, and the network vulnerability was lower than that of the CH gut microbial network (Figure 6A,B). In contrast, the robustness and vulnerability of the NCF and CF gut microbiota were not significantly different between the two water bodies. The N: P cohesion of the NCH gut microbiota was significantly higher than that of the CH gut microbiota (Figure 6C), and there was no significant difference in the N: P cohesion between the NCF and CF gut microbiota. Thus, there was a difference in the stability of the gut microbial community of *B. aeruginosa* between the two water conditions, whereas there was no significant difference in the stability of the gut microbial community of *P. canaliculata* .

### 3.6 Impact of Potential Keystone Taxa on the Gut Microbiota

**FIGURE 7:**
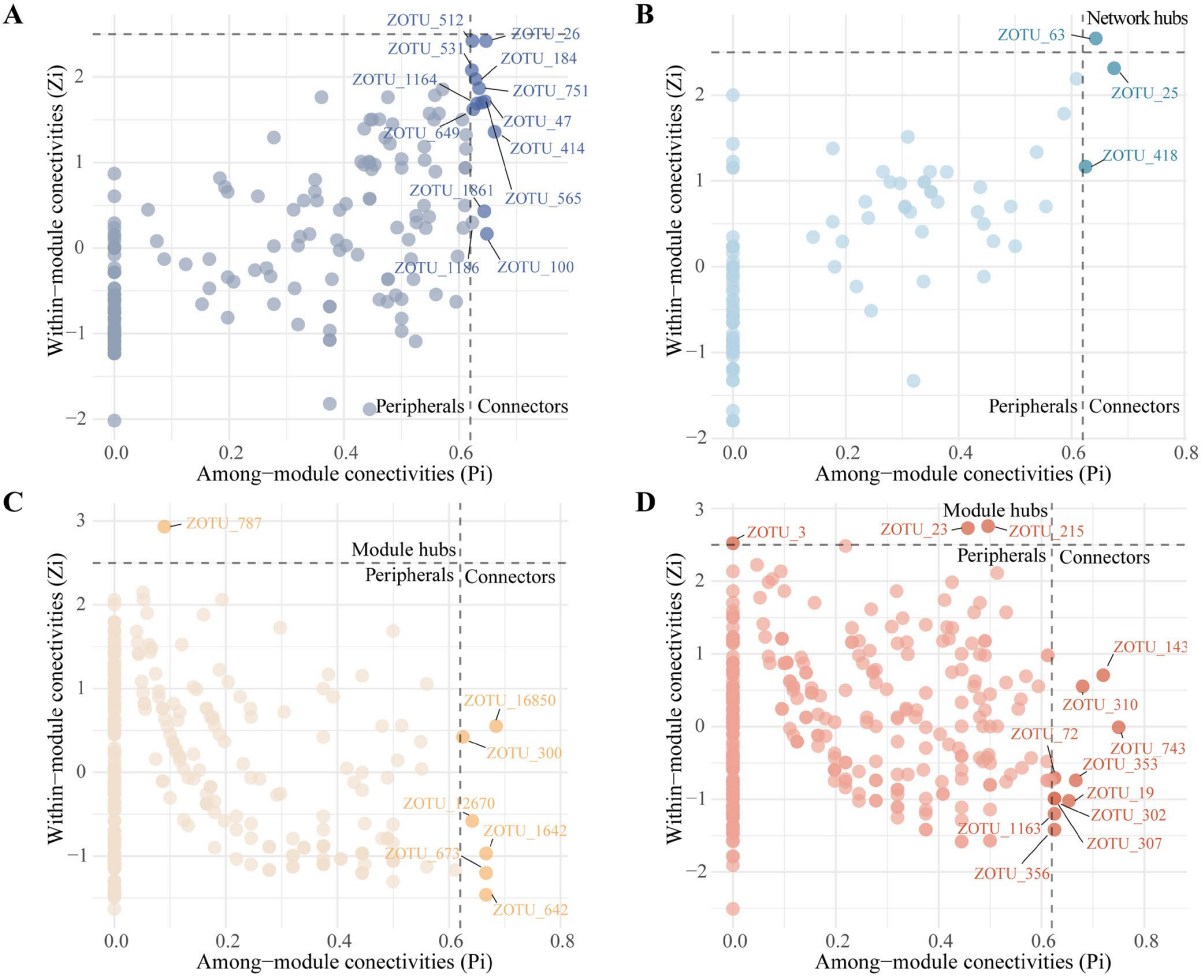
ZiPi showing the topological roles of NCH (A), CH (B), NCF (C), and CF (D) gut microbiota in the network, identifying potential keystones. The module hubs are identified as Zi ≥ 2.5, Pi < 0.62; the network hubs are identified as Zi ≥ 2.5, Pi ≥ 0.62; and the connectors are identified as Zi < 2.5, Pi ≥ 0.62. NCH: *B. aeruginosa* in WACB (*n* = 21); CH: *B. aeruginosa* in WCB (*n* = 21); NCF: *P. canaliculata* in WACB (*n* = 21); CF: *P. canaliculata* in WCB (*n* = 21).

Most OTUs in each network acted as peripherals in the gut microbial network (Figure 7, Table S5). There are thirteen connectors in the NCH gut microbial network from Firmicutes (ZOTU_512, ZOTU_531, ZOTU_751, ZOTU_1186, ZOTU_100, ZOTU_414, ZOTU_26), Bacteroidota (ZOTU_184, ZOTU_565), Actinobacteriota (ZOTU_649), Proteobacteria (ZOTU_47), and Unclassified (ZOTU_1164, ZOTU_1861). There are two connectors in the CH gut microbial network, both from Proteobacteria (ZOTU_25, ZOTU_418). There are six connectors in the NCF gut microbial network, including Proteobacteria (ZOTU_300, ZOTU_16850, ZOTU_12670, ZOTU_1642), Actinobacteria (ZOTU_673), and Proteobacteria (ZOTU_300). There are ten connectors in the CF gut microbial network from Proteobacteria (ZOTU_310, ZOTU_19, ZOTU_1163, ZOTU_143, ZOTU_743, ZOTU_353, ZOTU_307, ZOTU_72, ZOTU_302), and Cyanobacteria (ZOTU_356), and there are one and three module hubs in the NCF and CF gut microbial networks from Bacteroidota (ZOTU_787) and Proteobacteria (ZOTU_3, ZOTU_23, ZOTU_215), respectively. In addition, there was a network hub in the CH gut microbial network from Proteobacteria (ZOTU_63). To assess whether the keystone taxa contributed to the stabilization of the community, the keystone taxa were removed from the network, and the vulnerability of the network with the keystone taxa removed was calculated. We found that vulnerability changed after the removal of keystone taxa (Table S6). Across all the gut microbiota, the vulnerability of the network with keystone taxa removed was significantly higher than that of the original network, suggesting that the loss of keystone taxa destabilizes the network.

**FIGURE 8:**
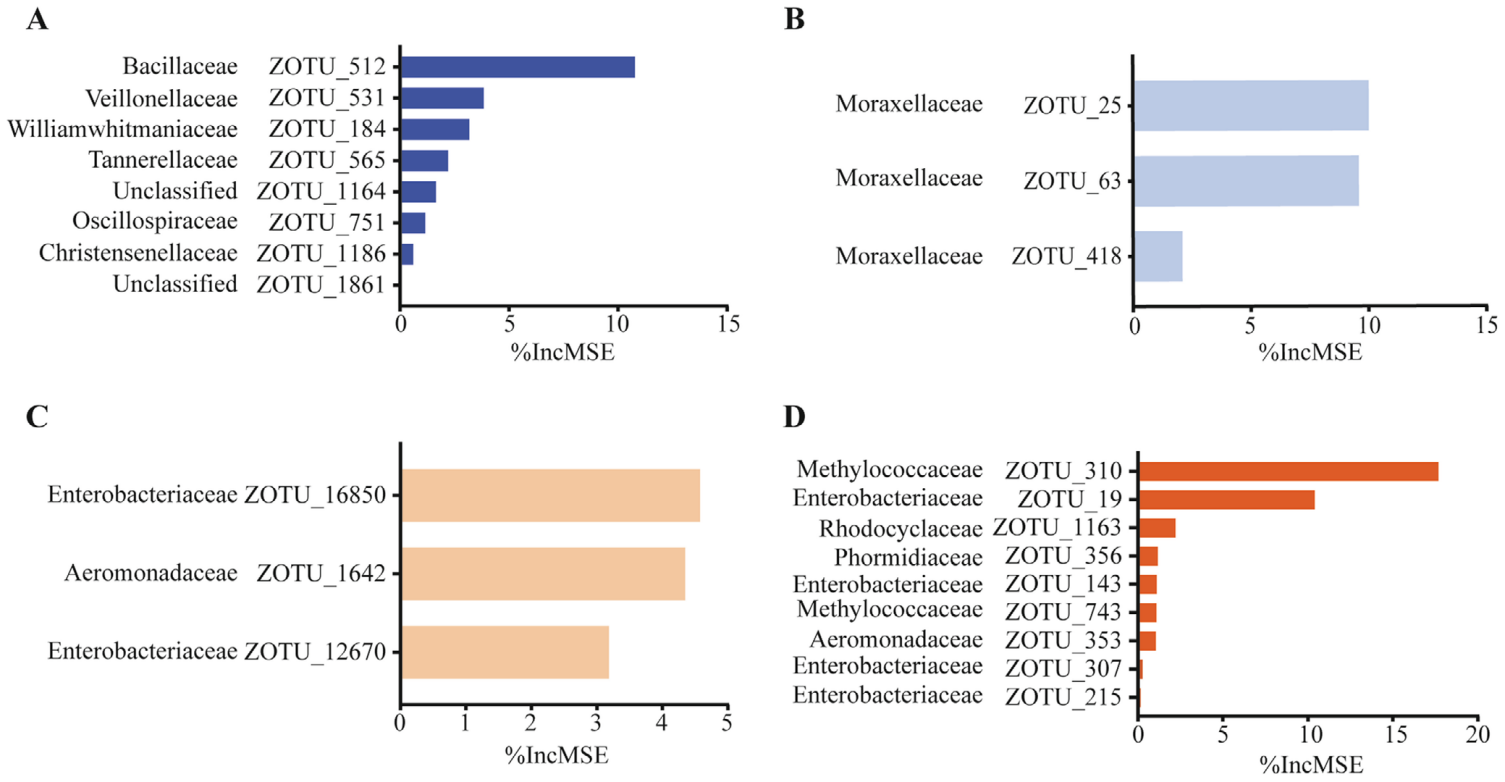
Random forest scores predicting the importance of potential keystones for community stabilization in the NCH (A), CH (B), NCF (C), and CF (D) gut microbiota. NCH: *B. aeruginosa* in WACB (*n* = 21); CH: *B. aeruginosa* in WCB (*n* = 21); NCF: *P. canaliculata* in WACB (*n* = 21); CF: *P. canaliculata* in WCB (*n* = 21).

Random forest analysis revealed that OTU_512 (Bacillaceae) and OTU_531 (Veillonellaceae) of the NCH gut microbiota were the most important keystones for predicting community stability, with 10.77% and 3.84% of community stability explained, respectively. In the CH gut microbiota, the important keystones predicting community stability were OTU_25 (Moraxellaceae) and OTU_63 (Moraxellaceae), which explained 10.01% and 9.59% of community stability, respectively. In the NCF gut microbiota, most of the keystones belonged to Enterobacteriaceae, with OTU_16850 (Enterobacteriaceae) and OTU_1642 (Aeromonadaceae) explaining 4.58% and 4.35% of the community stability, respectively. In the CF gut microbiota, OTU_310 (Methylococcaceae) and OTU_19 (Enterobacteriaceae) contributed the most to the prediction of community stability (Figure 8). In the gut microbial network of *B. aeruginosa*, there were 13 keystones in NCH and only 3 keystones in CH (Table S5), and the number of keystones among the gut microbiota of *B. aeruginosa* contributing to community stability was lower in WCB. In contrast, the gut microbial network of *P. canaliculata* had more keystones in WCB than in WACB, suggesting that it plays an important role in establishing network stability.

### 3.7 Effects of Biotic and Abiotic Factors on Community Stability

**FIGURE 9:**
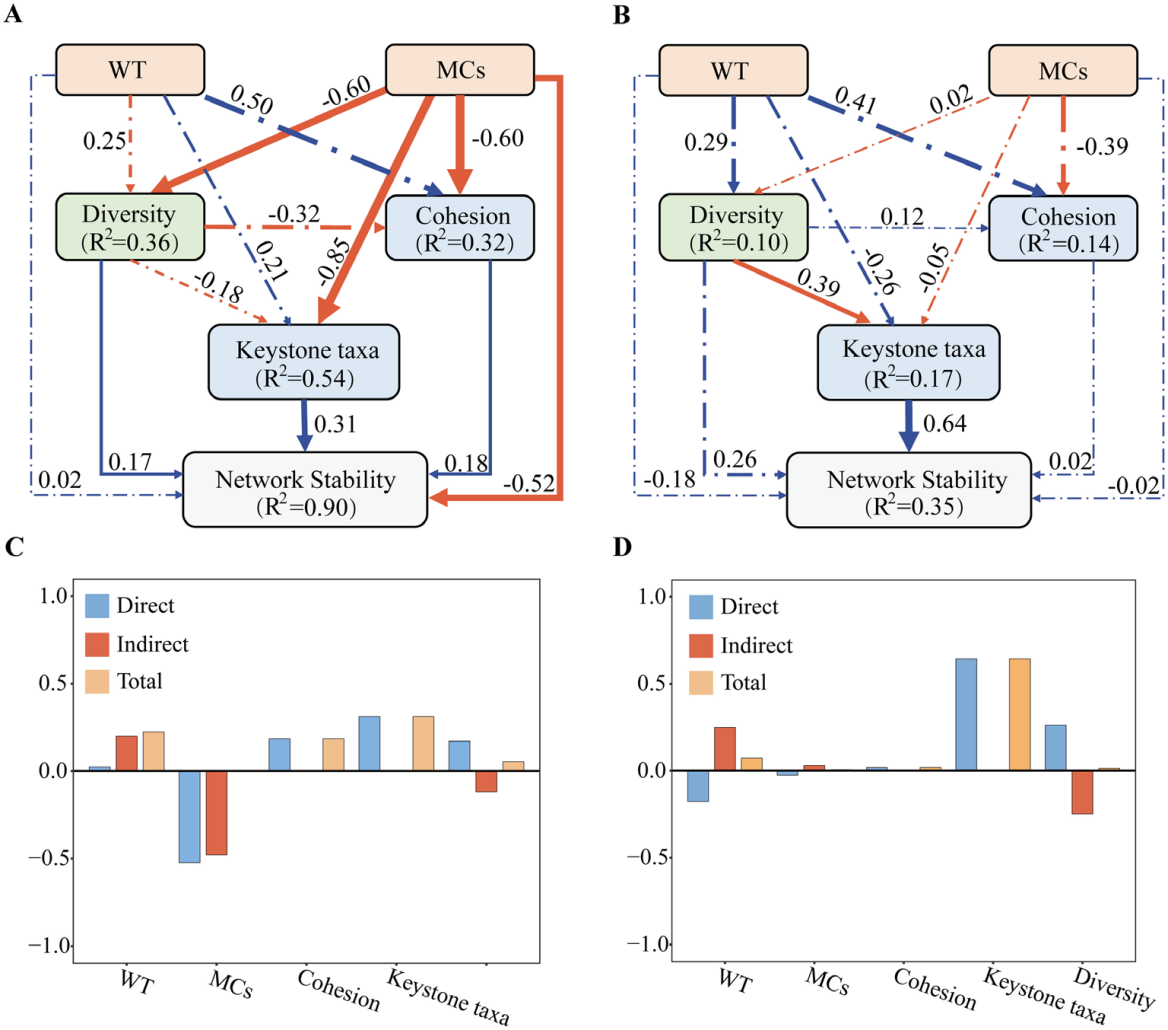
Piecewise structural equation modeling image showing factors affecting the stability of the gut microbial communities of (A) *B. aeruginosa* and (B) *P. canaliculata* . The solid blue lines indicate significant positive effects (*p* < 0.05), the solid red lines indicate significant negative effects (*p* < 0.05), and the dashed lines indicate non‐significant pathways. The width of each arrow is proportional to the standardized pathway coefficient. The numbers next to the arrows are standardized path coefficients, similar to relative regression weights, indicating the effect size of the relationship. Bars show standardized effects on network stability for the *B. aeruginosa* (C) and *P. canaliculata* (D) gut microbial communities.

Water temperature (WT) and MCs were screened as the main explanatory factors via a forward selection procedure. Piecewise structural equation modeling revealed that MCs had a significant negative effect on the network stability of the gut microbial community of *B. aeruginosa* (significant path coefficient = −0.52) (Figure 9A,C; Table S7), suggesting that network stability decreased in the presence of cyanobacterial toxins. MCs caused the diversity, cohesion, and keystone taxa in the gut microbial community of *B. aeruginosa* to significantly decrease in relative abundance (significant path coefficients = −0.60, −0.60 and −0.85). However, the diversity, cohesion and relative abundance of keystone taxa were positively correlated with network stability (significant path coefficients = 0.17, 0.18 and 0.31). Network stability showed a decreasing trend due to multiple drivers, with the MCs variable explaining significantly more changes in network stability than other influences. Whereas the gut microbial community of *P. canaliculata* was not significantly affected by WT and MCs (Figure 9B,D; Table S8), the network stability of the gut microbial community of *P. canaliculata* was positively correlated with keystone taxa (significant path coefficient = 0.64). All pathways accounted for 90% and 35% of the gut microbial network stability in *B. aeruginosa* and *P. canaliculata* , respectively.

## 4 Discussion

### 4.1 Differential Responses of Gut Microbial Community Composition in Two Gastropod Species to Cyanobacterial Blooms

This study revealed that at the phylum level, the gut microbial community compositions of the two snail species were similar, with the dominant phyla being Proteobacteria and Firmicutes. This suggests that the gut microbial communities of freshwater snails share some commonalities despite different host species, a pattern that has been demonstrated in several studies. The composition of the gut microbiota is very similar at the phylum level (Lyu et al. 2022; Hao et al. 2020). However, there were differences between species at the genus level. This may reflect differences in the recruitment of commensal microorganisms by different snail hosts, and many studies have also shown that gastropods living in the same habitat are host‐specific despite commonalities at some taxonomic levels. For instance, three species of snails living in the same freshwater ecosystem have more similar gut microbial compositions at the phylum level but greater differences at the genus level. Although the gut microbial communities of three snail species share the same core microbial community, the abundance of these core microbial communities varies significantly among the different snail species (Hu et al. 2024).

The same species also tends to have different microbial communities in different habitats, especially gastropods. For example, there were significant differences in the gut microbial composition of *P. canaliculata* under different temperature stresses (Li et al. 2022), which may indicate that the environment may more strongly influence the gut microbiota of snails. As a potent environmental stressor, toxic cyanobacterial blooms have been proven to affect the gut microbiota of *B. aeruginosa* (Lyu et al. 2022), and our results again provide further evidence that the gut microbiota of *P. canaliculata* appears unaffected by cyanobacterial blooms and exhibits a distinctly different response pattern. The gut microbiota of *P. canaliculata* has been shown to potentially contribute to its environmental adaptability. This is supported by the presence of unique dominant microbial communities such as Fibrobacteres and Tenericutes, which are more abundant in *P. canaliculata* (Zhou et al. 2022). Fibrobacteres are common primary degraders of cellulose in the intestines of herbivores, and Tenericutes are thought to be involved in carbohydrate storage, carbon fixation, and environmental responses, suggesting that this invasive snail may possess enhanced potential to adapt to novel environments. We found that *Mycoplasma* are highly abundant in the gut microbiota of *P. canaliculata* , and although the specific function of *Mycoplasma* in the mollusk gut is unknown, the presence of *Mycoplasma* in the gut has been shown to increase the metabolism of their fish hosts (Rasmussen et al. 2021). Thus, the gut microbiota of *P. canaliculata* is likely to have the capacity to increase their metabolism, which would improve their ability to adapt to environmental stresses.

### 4.2 Differential Responses of Gut Microbial Community Assembly in Two Gastropod Species to Cyanobacterial Blooms

The assembly of the gut microbiota of *P. canaliculata* is more stochastic than that of *B. aeruginosa*. Although previous studies have shown that the assembly of the gut microbiota of indigenous snails is more stochastic (Shi et al. 2024), the indigenous snails collected in their study belong to *Bellamya* and *Hemifusus*, which are more distantly phylogenetically related to each other in terms of host phylogeny (Shi et al. 2024), and this phylogenetic difference may be the reason for the greater stochasticity of the gut microbiota assembly process in the indigenous snails, which may further substantiate the critical role of host factors in the assembly of gut microbial communities. The balance between stochastic and deterministic processes in the assembly of snail gut microbes has been linked to host adaptation during snail acclimatization to new environments (Yuan et al. 2025). Thus, this difference in host species may also affect the response of snails to environmental stress. We found that the contribution of deterministic processes (homogeneous selection) to the assembly mechanism of the gut microbiota of *B. aeruginosa* in WCB was significantly higher than in WACB, which may be attributed to the fact that cyanobacteria provide a specific environmental condition and that the environmental perturbations may promote the growth and colonization of specific microbial communities, leading to its gut microbial community structure converged (Zhou and Ning 2017). The decrease in microbial diversity made it unable to defend itself effectively against external environmental disturbances, which led to greater sensitivity of the gut microbiota of *B. aeruginosa* to the cyanobacterial environment. In contrast, for *P. canaliculata* , stochastic processes (limited dispersal) contributed more to the gut microbiota in both WCB and WACB. Stochastic processes may increase resistance to external stresses during snail invasion and adaptation to new environments, thus maintaining a stable gut microbiota (Chase 2010), and stochastic processes play crucial roles in shaping the snail gut microbiota. In addition, unlike the filter‐feeding pathway used by *B. aeruginosa* (Qiu et al. 2017), *P. canaliculata* scrapes mainly through the radula (King‐Lun, Robert, and Jian‐Wen King‐Lun et al. 2009). Moreover, *P. canaliculata* is able to transition between different habitats in the water column and on land through its gills, trachea, and lung sacs, and these biological characteristics, which reduce its chances of ingesting cyanobacteria or being affected by cyanobacteria, might explain the lower response of its assembly mechanism to cyanobacteria.

### 4.3 Differential Responses of Gut Microbial Community Stability in Two Gastropod Species to Cyanobacterial Blooms

In this study, we found that the gut microbiota of *B. aeruginosa* exhibited low network complexity in WCB. However, *P. canaliculata* showed a stable network with high complexity in both WCB and WACB. Network complexity, as a key indicator of the interaction between microorganisms and their surroundings, can effectively reflect not only the stability of microbial communities (Yuan et al. 2021), but also the host's ability to adapt to environmental perturbations (Zhao et al. 2021). Microbial networks with relatively high complexity tend to be less susceptible to external environmental stresses than microbial networks with relatively low complexity (Santolini and Barabási 2018), and many studies have found that the gut microbes of aquatic animals exposed to various types of environmental stresses, such as the gut microbiota of zebrafish after exposure to silver nanoparticles and the gut microbiota of *Litopenaeus vannamei* under salt stress (Chen, Huang, et al. 2021; Deris et al. 2022), both present similar patterns. The differences in the stability of the gut microbiota of *P. canaliculata* and *B. aeruginosa* may further reflect the differences in their adaptation to the environment. In addition, attenuation of interspecies interactions usually reduces the coupling effects arising from strong cooperative interactions, thus promoting microbial community stabilization (Guo et al. 2022). N: P cohesion is an important measure of community stability, and higher negative cohesion usually means greater stability. Our study revealed lower N: P cohesion in the gut microbiota of *B. aeruginosa* in WCB, suggesting that cyanobacteria may enhance cooperative interactions among microbiota, thereby reducing the network stability of their gut microbiota.

Keystone taxa play a key role in maintaining network stability (Olesen et al. 2007). The removal of some potential keystones can affect interactions within the microbiota, leading to increased vulnerability and thus reduced network stability (Shi et al. 2016). Networks with higher complexity and more keystones tend to be more stable (Liu et al. 2022). In our study, the higher number of keystones possessed by the gut microbiota of *B. aeruginosa* in WCB than in WACB is consistent with the trend of network complexity and stability, suggesting that networks containing more keystones tend to be more stable, This may be since cyanobacteria reduce the gut microbial diversity of *B. aeruginosa* and thus its interspecific interactions and the number of keystones, leading to a decrease in the stability of its network. Notably, in the NCH gut microbiota, most of the keystones are from Firmicutes, which can maintain the stability of the gut microbiome by breaking down complex carbohydrates and dietary fibers (Stojanov et al. 2020). Among them, the Bacillaceae are key in maintaining the stability of gut microorganisms. Bacillaceae have antimicrobial activity and are able to maintain host health by colonizing the host gut and producing abundant digestive enzymes that inhibit pathogenic microorganisms (Wang et al. 2021). The keystones of the CH gut microbiome are all from the family Moraxellaceae of Proteobacteria. Moraxellaceae encompasses various opportunistic pathogens (Teixeira and Merquior 2014), but in our study, its role as a keystone in maintaining intestinal stability might be due to its ability to degrade microcystins produced by cyanobacteria (Morón‐López et al. 2020). In addition, the keystones of *P. canaliculata* gut microbiota in WCB and WACB are similar in that Enterobacteriaceae of Proteobacteria dominates both to maintain gut microbial stability, which may indirectly promote host health by preventing pathogenic bacteria from establishing or proliferating (Zhang et al. 2021), which may explain the stabilization of the gut microbiota of *P.* *canaliculata*.

## 5 Conclusion

In water bodies with occurrences of cyanobacterial bloom, the diversity of gut microorganisms was significantly lower in *B. aeruginosa*, with a high proportion of homogeneous selection processes in its community assembly, significantly weaker network complexity and stability, and a reduced number of keystones. In contrast, the gut microbiota of *P. canaliculata* maintained high network complexity and stability in the cyanobacterial environment, and stochastic processes dominated its community assembly, and keystones continued to play a regulatory role to maintain the stability of the gut microbial community. These findings suggest that the gut microbiota of *B. aeruginosa* and *P. canaliculata* exhibit distinct response patterns in the face of cyanobacterial bloom stress. These results not only contribute to the understanding of the host‐specific response mechanisms of gastropods to environmental perturbations but also may provide important information to unravel the invasion mechanism of *P. canaliculata* .

## Author Contributions

**Kexin Meng:** data curation (lead), formal analysis (lead), investigation (equal), visualization (lead), writing – original draft (lead), writing – review and editing (equal). **Wen Yang:** investigation (equal), methodology (equal), writing – review and editing (equal). **Shuangye Pan:** resources (equal), software (equal). **Kaihong Lu:** project administration (equal). **Jinyong Zhu:** conceptualization (equal), funding acquisition (lead), project administration (equal), supervision (equal), writing – review and editing (equal).

## Conflicts of Interest

The authors declare no conflicts of interest.

## Supporting information


**Table S1:** Summary of changes in environmental variables at different sampling times for the pond with the absence of cyanobacterial bloom and the pond with occurrences of cyanobacterial bloom (mean ± SD). “‐” indicates that the SD value has exceeded the water depth.
**Table S2:** Phytoplankton biomass in the pond with the absence of cyanobacterial bloom and the pond with occurrences of cyanobacterial bloom during different sampling months (mg/L).
**Table S3:** ANOSIM analysis between different gut microbiota. NCH: *B. aeruginosa* in WACB (*n* = 21); CH: *B. aeruginosa* in WCB (*n* = 21); NCF: *P. canaliculata* in WACB (*n* = 21); CF: *P. canaliculata* in WCB (*n* = 21).
**Table S4:** Topological parameters of the gut co‐occurrence network of *B. aeruginosa* and *P. canaliculata* in WCB and WACB. NCH: *B. aeruginosa* in WACB (*n* = 21); CH: *B. aeruginosa* in WCB (*n* = 21); NCF: *P. canaliculata* in WACB (*n* = 21); CF: *P. canaliculata* in WCB (*n* = 21).
**Table S5:** Random forest analysis was used to predict the importance of the mean square error (MSE) keystones for community stability. NCH: *B. aeruginosa* in WACB (*n* = 21); CH: *B. aeruginosa* in WCB (*n* = 21); NCF: *P. canaliculata* in WACB (*n* = 21); CF: *P. canaliculata* in WCB (*n* = 21).
**Table S6:** Comparison of the gut microbial community vulnerability of *B. aeruginosa* and *P. canaliculata* in WCB and WACB. NCH: *B. aeruginosa* in WACB (*n* = 21); CH: *B. aeruginosa* in WCB (*n* = 21); NCF: *P. canaliculata* in WACB (*n* = 21); CF: *P. canaliculata* in WCB (*n* = 21).
**Table S7:** Direct and indirect effects between different latent variables on the gut microbial community of *B. aeruginosa*.
**Table S8:** Direct and indirect effects between different latent variables on the gut microbial community of *P. canaliculata* .

## Data Availability

Raw sequence data presented in the study are deposited in the NCBI repository, accession number PRJNA1268002.
