# Supplementary material for: Divergent Response of Host‐Specific Driven Gut Microbial Stability in Freshwater Gastropods to Cyanobacterial Blooms
Source: Ecol Evol. 2025 Nov 18;15(11):e72541. doi: 10.1002/ece3.72541 (PMC12626645; doi:10.1002/ece3.72541)
Supplement: Supplementary file 1 — Table S1: Summary of changes in environmental variables at different sampling times for the pond with the absence of cyanobacterial bloom and the pond with occurrences of cyanobacterial bloom (mean ± SD). “‐” indicates that the SD value has exceeded the water depth. Table S2: Phytoplankton biomass in the pond with the absence of cyanobacterial bloom and the pond with occurrences of cyanobacterial bloom during different sampling months (mg/L). Table S3: ANOSIM analysis between different gut microbiota. NCH: B. aeruginosa in WACB (n = 21); CH: B. aeruginosa in WCB (n = 21); NCF: P. canaliculata in WACB (n = 21); CF: P. canaliculata in WCB (n = 21). Table S4: Topological parameters of the gut co‐occurrence network of B. aeruginosa and P. canaliculata in WCB and WACB. NCH: B. aeruginosa in WACB (n = 21); CH: B. aeruginosa in WCB (n = 21); NCF: P. canaliculata in WACB (n = 21); CF: P. canaliculata in WCB (n = 21). Table S5: Random forest analysis was used to predict the importance of the mean square error (MSE) keystones for community stability. NCH: B. aeruginosa in WACB (n = 21); CH: B. aeruginosa in WCB (n = 21); NCF: P. canaliculata in WACB (n = 21); CF: P. canaliculata in WCB (n = 21). Table S6: Comparison of the gut microbial community vulnerability of B. aeruginosa and P. canaliculata in WCB and WACB. NCH: B. aeruginosa in WACB (n = 21); CH: B. aeruginosa in WCB (n = 21); NCF: P. canaliculata in WACB (n = 21); CF: P. canaliculata in WCB (n = 21). Table S7: Direct and indirect effects between different latent variables on the gut microbial community of B. aeruginosa. Table S8: Direct and indirect effects between different latent variables on the gut microbial community of P. canaliculata . [file ECE3-15-e72541-s001.docx]

**Supplemental Tables:**

**Table S1. Summary of changes in environmental variables at different sampling times for** **the pond with the absence of cyanobacterial bloom and** **the pond with occurrences of cyanobacterial bloom** **(mean ± SD). “-” indicates that the SD value could not be measured due to the shallow depth of the water body.**

|  | May | Jun. | Jul. | Aug. | Sept. | Oct. | Nov. |
| --- | --- | --- | --- | --- | --- | --- | --- |
| **Pond with the absence** **of cyanobacterial bloom** | | | | | | | |
| WT (°C) | 24.5 | 26.7 | 27.7 | 29.8 | 24.6 | 21.6 | 16.9 |
| DO (mg/L) | 6.81±0.03 | 5.24±0.02 | 4.13±0.04 | 5.56±0.03 | 4.35±0.03 | 9.32±0.04 | 7.26±0.03 |
| SD (cm) | - | - | - | - | - | - | - |
| pH | 7.85±0.02 | 7.42±0.01 | 7.30±0.10 | 7.95±0.12 | 7.12±0.05 | 6.85±0.06 | 7.53±0.03 |
| NH_4_N (mg/L) | 0.19±0.02 | 0.15±0.01 | 0.09±0.01 | 0.12±0.01 | 0.25±0.02 | 0.21±0.01 | 0.24±0.03 |
| NO_2_N (mg/L) | 0.048±0.002 | 0.039±0.001 | 0.022±0.005 | 0.035±0.008 | 0.067±0.015 | 0.062±0.003 | 0.049±0.002 |
| NO_3_N (mg/L) | 0.602±0.007 | 0.415±0.005 | 0.302±0.006 | 0.587±0.025 | 0.924±0.085 | 0.765±0.007 | 0.823±0.028 |
| TN (mg/L) | 0.955±0.018 | 0.682±0.015 | 0.715±0.032 | 0.914±0.075 | 1.326±0.201 | 1.088±0.077 | 1.152±0.142 |
| PO_4_P (mg/L) | 0.072±0.006 | 0.091±0.004 | 0.112±0.005 | 0.131±0.008 | 0.067±0.003 | 0.085±0.010 | 0.054±0.002 |
| TP (mg/L) | 0.078±0.003 | 0.095±0.011 | 0.121±0.002 | 0.134±0.008 | 0.073±0.004 | 0.086±0.015 | 0.069±0.029 |
| Chl-a (µg//L) | 5.32±0.07 | 9.85±0.15 | 12.47±0.11 | 18.24±0.04 | 8.13±0.06 | 4.92±0.08 | 3.87±0.05 |
| **Pond with occurrences of cyanobacterial bloom** | | | | | | | |
| WT (°C) | 24.7 | 26.2 | 26.9 | 29.3 | 25.8 | 22.8 | 17.4 |
| DO (mg/L) | 6.36±0.04 | 3.66±0.01 | 1.49±0.01 | 3.82±0.09 | 1.57±0.02 | 9.14±0.16 | 8.58±0.13 |
| SD (cm) | 38.90±0.49 | 71±0.82 | 60.00±1.63 | 17.4±0.22 | 27.00±2.45 | 56±0.82 | 46±0.82 |
| pH | 8.37±0.03 | 7.19±0.02 | 7.13±0.14 | 8.51±0.14 | 6.90±0.07 | 6.23±0.08 | 7.64±0.02 |
| NH_4_N (mg/L) | 0.22±0.03 | 0.07±0.01 | 0.04±0.01 | 0.10±0.01 | 0.30±0.01 | 0.26±0.01 | 0.28±0.04 |
| NO_2_N (mg/L) | 0.043±0.004 | 0.034±0.003 | 0.018±0.007 | 0.028±0.012 | 0.083±0.021 | 0.075±0.002 | 0.053±0.004 |
| NO_3_N (mg/L) | 0.521±0.008 | 0.307±0.003 | 0.278±0.007 | 0.614±0.029 | 1.037±0.091 | 0.890±0.008 | 0.898±0.031 |
| TN (mg/L) | 0.872±0.155 | 0.515±0.004 | 0.604±0.037 | 0.829±0.082 | 1.491±0.216 | 1.002±0.082 | 1.264±0.155 |
| PO_4_P (mg/L) | 0.099±0.008 | 0.085±0.003 | 0.121±0.006 | 0.145±0.010 | 0.058±0.004 | 0.071±0.013 | 0.049±0.003 |
| TP (mg/L) | 0.099±0.008 | 0.089±0.012 | 0.127±0.001 | 0.147±0.009 | 0.061±0.005 | 0.832±0.018 | 0.071±0.032 |
| Chl-a (µg/L) | 27.65±0.41 | 11.92±0.18 | 8.92±0.09 | 23.19±0.03 | 14.91±0.08 | 6.89±0.09 | 4.54±0.06 |

**Table S2.** **Phytoplankton biomass in the pond with the absence of cyanobacterial bloom and the pond with occurrences of cyanobacterial bloom during different sampling months (mg/L).**

|  | May | | Jun. | | Jul. | | Aug. | | Sep. | | Oct. | | Nov. | |
| --- | --- | --- | --- | --- | --- | --- | --- | --- | --- | --- | --- | --- | --- | --- |
| **Pond with the absence of cyanobacterial bloom** | | | | | | | | | | | | | | |
| Cyanophyta | 0.510 | | 0 | | 8.418 | | 7.670 | | 0.173 | | 0.000 | | 0.100 | |
| Bacillariophyta | 1.290 | | 0 | | 0 | | 1.860 | | 0.465 | | 0.470 | | 0.770 | |
| Cryptophyta | 0 | | 0 | | 0 | | 5.130 | | 3.600 | | 0 | | 0 | |
| Pyrrophyta | 0 | | 0 | | 21.712 | | 36.673 | | 0.234 | | 0 | | 0.635 | |
| Euglenophyta | 5.690 | | 11.580 | | 20.665 | | 32.700 | | 22.202 | | 22.130 | | 27.300 | |
| Chlorophyta | 0.500 | | 1.630 | | 1.361 | | 3.490 | | 7.884 | | 35.210 | | 32.725 | |
| **Pond with occurrences of cyanobacterial bloom** | | | | | | | | | | | | | | |
| Cyanophyta | | 165.717 | | 38.019 | | 4.240 | | 92.959 | | 26.289 | | 7.615 | | 3.964 |
| Bacillariophyta | | 0.135 | | 0.127 | | 0.271 | | 1.885 | | 0.453 | | 1.355 | | 0.471 |
| Chrysophyta | | 0 | | 0 | | 1.088 | | 0 | | 1.088 | | 0 | | 0 |
| Cryptophyta | | 0.631 | | 0.842 | | 0.631 | | 0.421 | | 0.631 | | 2.104 | | 1.052 |
| Pyrrophyta | | 0 | | 0 | | 0 | | 0 | | 0 | | 9.001 | | 16.210 |
| Euglenophyta | | 2.099 | | 2.252 | | 10.776 | | 7.705 | | 2.252 | | 5.650 | | 22.576 |
| Chlorophyta | | 13.675 | | 4.384 | | 5.160 | | 10.441 | | 4.617 | | 13.633 | | 18.918 |

**Table S3. ANOSIM analysis between different gut microbiota. NCH: *B. aeruginosa* in WACB (n = 21); CH: *B. aeruginosa* in WCB (n = 21); NCF: *P. canaliculata* in WACB (n = 21); CF: *P. canaliculata* in WCB (n = 21).**

| **Groups** | **F** | ***R*²** | ***P* value** |
| --- | --- | --- | --- |
| NCH vs CH | 14.014 | 0.259 | 0.001 |
| NCH vs NCF | 13.377 | 0.251 | 0.001 |
| NCH vs CF | 11.874 | 0.229 | 0.001 |
| CH vs NCF | 25.210 | 0.387 | 0.001 |
| CH vs CF | 21.949 | 0.354 | 0.001 |
| NCF vs CF | 1.207 | 0.029 | 0.214 |

**Table S4 Topological parameters of the gut co-occurrence network of *B. aeruginosa* and *P. canaliculata* in WCB and WACB. NCH: *B. aeruginosa* in WACB (n = 21); CH: *B. aeruginosa* in WCB (n = 21); NCF: *P. canaliculata* in WACB (n = 21); CF: *P. canaliculata* in WCB (n = 21).**

| Network Index | NCH | CH | NCF | CF |
| --- | --- | --- | --- | --- |
| Nodes | 184 | 108 | 309 | 356 |
| Edges | 1833 | 525 | 2439 | 2030 |
| Average degree | 19.9239 | 9.7222 | 15.7864 | 11.4045 |
| Average path length | 1.8015 | 2.0087 | 2.3497 | 2.7315 |
| Network diameter | 6.3283 | 6.0772 | 5.5710 | 8.2795 |
| Network density | 0.1089 | 0.0909 | 0.0513 | 0.032125 |
| Clustering coefficient | 0.5550 | 0.5985 | 0.5976 | 0.4890 |
| Modularity | 0.3254 | 0.4819 | 0.6450 | 0.6170 |
| Betweenness centralization | 0.0505 | 0.0768 | 0.0591 | 0.0748 |
| Closeness centralization | 1.2177 | 1.1839 | 0.1548 | 1.4530 |
| connectance | 0.1089 | 0.0909 | 0.0513 | 0.0321 |

**Table S5. Random forest analysis was used to predict the importance of the mean square error (MSE) keystones for community stability. NCH: *B. aeruginosa* in WACB (n = 21); CH: *B. aeruginosa* in WCB (n = 21); NCF: *P. canaliculata* in WACB (n = 21); CF: *P. canaliculata* in WCB (n = 21).**

| OTU ID | | Phylum | | Class | Order | Family | Genus | Role | Relative abundance | Increase in MSE (%) |  |  |  |  |  |
| --- | --- | --- | --- | --- | --- | --- | --- | --- | --- | --- | --- | --- | --- | --- | --- |
|  | **NCH** | | | | | | | | | |  |  |  |  |  |
| ZOTU_512 | | Firmicutes | | Bacilli | Bacillales | Bacillaceae | *Geobacillus* | connector | 0.000533 | 10.77 |  |  |  |  |  |
| ZOTU_531 | | Firmicutes | | Negativicutes | Veillonellales-Selenomonadales | Veillonellaceae | *Dialister* | connector | 0.000484 | 3.84 |  |  |  |  |  |
| ZOTU_184 | | Bacteroidota | | Bacteroidia | Bacteroidales | Williamwhitmaniaceae | *BCf9-17_termite_group* | connector | 0.000109 | 3.18 |  |  |  |  |  |
| ZOTU_565 | | Bacteroidota | | Bacteroidia | Bacteroidales | Tannerellaceae | *Parabacteroides* | connector | 0.000470 | 2.2 |  |  |  |  |  |
| ZOTU_1164 | | Unclassified | | Unclassified | Unclassified | Unclassified | *Unclassified* | connector | 0.000205 | 1.65 |  |  |  |  |  |
| ZOTU_751 | | Firmicutes | | Clostridia | Oscillospirales | Oscillospiraceae | *Flavonifractor* | connector | 0.000274 | 1.16 |  |  |  |  |  |
| ZOTU_1186 | | Firmicutes | | Clostridia | Christensenellales | Christensenellaceae | *Christensenellaceae_R-7_group* | connector | 0.000510 | 0.61 |  |  |  |  |  |
| ZOTU_1861 | | Unclassified | | Unclassified | Unclassified | Unclassified | *Unclassified* | connector | 0.000125 | 0.03 |  |  |  |  |  |
| ZOTU_100 | | Firmicutes | | Unclassified | Unclassified | Unclassified | *Unclassified* | connector | 0.000124 | -1.53 |  |  |  |  |  |
| ZOTU_414 | | Firmicutes | | Negativicutes | Acidaminococcales | Acidaminococcaceae | *Phascolarctobacterium* | connector | 0.001087 | -1.55 |  |  |  |  |  |
| ZOTU_26 | | Firmicutes | | Unclassified | Unclassified | Unclassified | *Unclassified* | connector | 0.000355 | -3.15 |  |  |  |  |  |
| ZOTU_649 | | Actinobacteriota | | Actinobacteria | Bifidobacteriales | Bifidobacteriaceae | *Bifidobacterium* | connector | 0.000618 | -3.27 |  |  |  |  |  |
| ZOTU_47 | | Proteobacteria | | Gammaproteobacteria | Enterobacterales | Enterobacteriaceae | *Unclassified* | connector | 0.000517 | -6.28 |  |  |  |  |  |
|  | **CH** | | | | | | | | | |  |  |  |  |  |
| ZOTU_25 | | Proteobacteria | | Gammaproteobacteria | Pseudomonadales | Moraxellaceae | *Acinetobacter* | connector | 0.010548 | 10.01 |  |  |  |  |  |
| ZOTU_63 | | Proteobacteria | | Gammaproteobacteria | Pseudomonadales | Moraxellaceae | *Acinetobacter* | network hub | 0.00669 | 9.59 |  |  |  |  |  |
| ZOTU_418 | | Proteobacteria | | Gammaproteobacteria | Pseudomonadales | Moraxellaceae | *Acinetobacter* | connector | 0.000516 | 2.09 |  |  |  |  |  |
|  | **NCF** | | | | | | | | | |  |  |  |  |  |
| ZOTU_16850 | | | Proteobacteria | Gammaproteobacteria | Enterobacterales | Enterobacteriaceae | *Unclassified* | connector | 0.000166 | 4.58 |  |  |  |  |  |
| ZOTU_1642 | | | Proteobacteria | Gammaproteobacteria | Aeromonadales | Aeromonadaceae | *Aeromonas* | connector | 0.000142 | 4.35 |  |  |  |  |  |
| ZOTU_12670 | | | Proteobacteria | Gammaproteobacteria | Enterobacterales | Enterobacteriaceae | *Unclassified* | connector | 0.000630 | 3.19 |  |  |  |  |  |
| ZOTU_787 | | | Bacteroidota | Bacteroidia | Bacteroidales | Unclassified | *Unclassified* | module hub | 0.000492 | -0.75 |  |  |  |  |  |
| ZOTU_673 | | | Actinobacteriota | Acidimicrobiia | Microtrichales | Ilumatobacteraceae | *CL500-29_marine_group* | connector | 0.000200 | -1.09 |  |  |  |  |  |
| ZOTU_642 | | | Firmicutes | Unclassified | Unclassified | Unclassified | *Unclassified* | connector | 0.000367 | -3.18 |  |  |  |  |  |
| ZOTU_300 | | | Proteobacteria | Gammaproteobacteria | Pseudomonadales | Pseudomonadaceae | *Pseudomonas* | connector | 0.00015 | -3.77 |  |  |  |  |  |
|  | **CF** | | | | | | | | | |  |  |  |  | connector |
| ZOTU_310 | | Proteobacteria | | Gammaproteobacteria | Methylococcales | Methylococcaceae | *Methyloparacoccus* | connector | 0.000731 | 17.68 |  |  |  |  |  |
| ZOTU_19 | | Proteobacteria | | Gammaproteobacteria | Enterobacterales | Enterobacteriaceae | *Unclassified* | connector | 0.014965 | 10.40 |  |  |  |  |  |
| ZOTU_1163 | | Proteobacteria | | Gammaproteobacteria | Burkholderiales | Rhodocyclaceae | *Uliginosibacterium* | connector | 0.000167 | 2.21 |  |  |  |  |  |
| ZOTU_356 | | Cyanobacteria | | Cyanobacteriia | Cyanobacteriales | Phormidiaceae | *Unclassified* | connector | 0.001461 | 1.17 |  |  |  |  |  |
| ZOTU_143 | | Proteobacteria | | Gammaproteobacteria | Enterobacterales | Enterobacteriaceae | *Citrobacter* | connector | 0.000418 | 1.11 |  |  |  |  |  |
| ZOTU_743 | | Proteobacteria | | Gammaproteobacteria | Methylococcales | Methylococcaceae | *Unclassified* | connector | 0.000113 | 1.10 |  |  |  |  |  |
| ZOTU_353 | | Proteobacteria | | Gammaproteobacteria | Aeromonadales | Aeromonadaceae | *Aeromonas* | connector | 0.001546 | 1.05 |  |  |  |  |  |
| ZOTU_307 | | Proteobacteria | | Gammaproteobacteria | Enterobacterales | Enterobacteriaceae | *Unclassified* | connector | 0.000156 | 0.28 |  |  |  |  |  |
| ZOTU_215 | | Proteobacteria | | Gammaproteobacteria | Enterobacterales | Enterobacteriaceae | *Plesiomonas* | module hub | 0.003675 | 0.17 |  |  |  |  |  |
| ZOTU_3 | | Proteobacteria | | Gammaproteobacteria | Xanthomonadales | Xanthomonadaceae | *Lysobacter* | module hub | 0.006219 | -1.07 |  |  |  |  |  |
| ZOTU_72 | | Proteobacteria | | Gammaproteobacteria | Enterobacterales | Enterobacteriaceae | *Unclassified* | connector | 0.001946 | -1.32 |  |  |  |  |  |
| ZOTU_23 | | Proteobacteria | | Gammaproteobacteria | Enterobacterales | Enterobacteriaceae | *Unclassified* | module hub | 0.011041 | -2.17 |  |  |  |  |  |
| ZOTU_302 | | Proteobacteria | | Alphaproteobacteria | Rhizobiales | Beijerinckiaceae | *Methylobacterium-Methylorubrum* | connector | 0.000125 | -2.71 |  |  |  |  |  |

**Table S6. Comparison of the gut microbial community vulnerability of *B. aeruginosa* and *P. canaliculata* in WCB and WACB. NCH: *B. aeruginosa* in WACB (n = 21); CH: *B. aeruginosa* in WCB (n = 21); NCF: *P. canaliculata* in WACB (n = 21); CF: *P. canaliculata* in WCB (n = 21).**

|  | NCH | CH | NCF | CF |
| --- | --- | --- | --- | --- |
| Unremoved of keystones | 0.0485 | 0.1520 | 0.0071 | 0.0060 |
| Removed of keystones | 0.0772 | 0.2756 | 0.0180 | 0.0129 |

**Table S7. Direct and indirect effects between different latent variables on the gut microbial community of *B. aeruginosa*.**

| Relationships | Direct | Indirect | Total |
| --- | --- | --- | --- |
| WT → Cohesion | 0.48 | 0.00 | 0.48 |
| WT → Keystone taxa | 0.22 | 0.00 | 0.22 |
| WT → Diversity | 0.26 | 0.00 | 0.26 |
| WT → Network Stability | 0.02 | 0.20 | 0.22 |
| Cohesion → Network Stability | 0.18 | 0.00 | 0.18 |
| Keystone taxa → Network Stability | 0.31 | 0.00 | 0.31 |
| MCs → Cohesion | -0.60 | 0.00 | -0.60 |
| MCs → Keystone taxa | -0.85 | 0.00 | -0.85 |
| MCs → Diversity | -0.60 | 0.00 | -0.60 |
| MCs → Network Stability | -0.52 | -0.48 | -1.00 |
| Diversity → Cohesion | -0.32 | 0.00 | -0.32 |
| Diversity → Keystone taxa | -0.18 | 0.00 | -0.18 |
| Diversity → Network Stability | 0.17 | -0.12 | 0.05 |

**Table S8. Direct and indirect effects between different latent variables on the gut microbial community of *P. canaliculata*.**

| Relationships | Direct | Indirect | Total |
| --- | --- | --- | --- |
| WT → Cohesion | 0.41 | 0.00 | 0.41 |
| WT → Keystone taxa | -0.26 | 0.00 | -0.26 |
| WT → Diversity | 0.30 | 0.00 | 0.30 |
| WT → Network Stability | -0.18 | 0.25 | 0.07 |
| Cohesion → Network Stability | 0.02 | 0.00 | 0.02 |
| Keystone taxa → Network Stability | 0.64 | 0.00 | 0.64 |
| MCs → Cohesion | -0.39 | 0.00 | -0.39 |
| MCs → Keystone taxa | -0.05 | 0.00 | -0.05 |
| MCs → Diversity | 0.02 | 0.00 | 0.02 |
| MCs →Network Stability | -0.02 | 0.03 | 0.01 |
| Diversity →Cohesion | 0.12 | 0.00 | 0.12 |
| Diversity →Keystone taxa | 0.39 | 0.00 | 0.39 |
| Diversity →Network Stability | 0.26 | -0.25 | 0.01 |
